# Bringing to Light the Importance of the miRNA Methylome in Colorectal Cancer Prognosis Through Electrochemical Bioplatforms

**DOI:** 10.1021/acs.analchem.3c05474

**Published:** 2024-02-13

**Authors:** Eloy Povedano, Víctor Ruiz-Valdepeñas Montiel, Ravery Sebuyoya, Rebeca M. Torrente-Rodríguez, Maria Garranzo-Asensio, Ana Montero-Calle, José M. Pingarrón, Rodrigo Barderas, Martin Bartosik, Susana Campuzano

**Affiliations:** † Departamento de Química Analítica, Facultad de CC. Químicas, Universidad Complutense de Madrid, Pza. de las Ciencias 2, Madrid 28040, Spain; ‡ Research Centre for Applied Molecular Oncology, Masaryk Memorial Cancer Institute, Zluty kopec 7, Brno 656 53, Czech Republic; § National Centre for Biomolecular Research, Faculty of Science, Masaryk University, Kamenice 5, Brno 625 00, Czech Republic; ∥ Chronic Disease Programme, UFIEC, Institute of Health Carlos III, Majadahonda, Madrid 28220, Spain

## Abstract

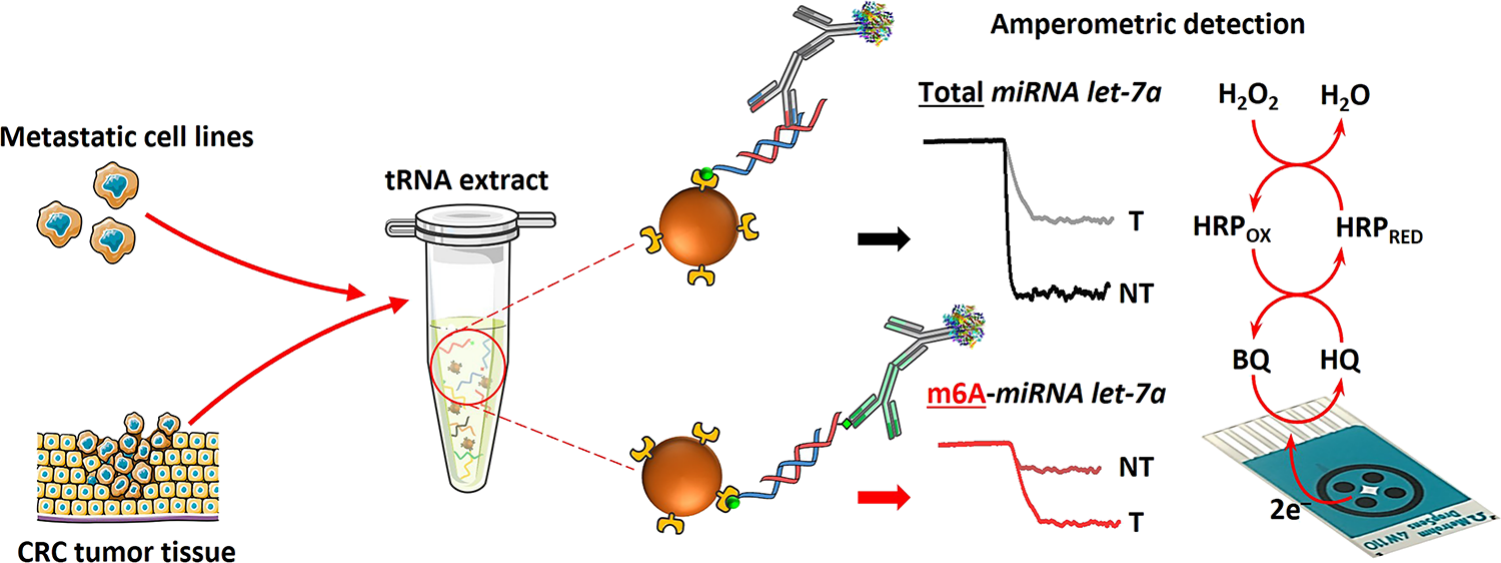

This work reports the first electrochemical bioplatforms developed for the determination of the total contents of either target miRNA or methylated target miRNA. The bioplatforms are based on the hybridization of the target miRNA with a synthetic biotinylated DNA probe, the capture of the formed DNA/miRNA heterohybrids on the surface of magnetic microcarriers, and their recognition with an antibody selective to these heterohybrids or to the *N*^6^-methyladenosine (m6A) epimark. The determination of the total or methylated target miRNA was accomplished by labeling such secondary antibodies with the horseradish peroxidase (HRP) enzyme. In both cases, amperometric transduction was performed on the surface of disposable electrodes after capturing the resulting HRP-tagged magnetic bioconjugates. Because of their increasing relevance in colorectal cancer (CRC) diagnosis and prognosis, miRNA let-7a and m6A methylation were selected. The proposed electrochemical bioplatforms showed attractive analytical and operational characteristics for the determination of the total and m6A-methylated target miRNA in less than 75 min. These bioplatforms, innovative in design and application, were applied to the analysis of total RNA samples extracted from cultured cancer cells with different metastatic profiles and from paired healthy and tumor tissues of patients diagnosed with CRC at different stages. The obtained results demonstrated, for the first time using electrochemical platforms, the potential of interrogating the target miRNA methylation level to discriminate the metastatic capacities of cancer cells and to identify tumor tissues and, in a pioneering way, the potential of the m6A methylation in miRNA let-7a to serve as a prognostic biomarker for CRC.

Colorectal cancer (CRC) is one of the leading causes of cancer death worldwide. Despite the constant advances in the diagnosis and management of patients, several gaps remain to be addressed, from early detection to identification of prognostic variables, effective treatment of metastatic disease, and implementation of personalized treatment strategies. The prognosis of advanced CRC continues to be bleak, and thus, the search for prognostic and predictive biomarkers to guide physicians in the management and treatment of this disease is considered an urgent demand.

Recently, biomarkers associated with the epigenome of tumors have gained more attention.^1−3^ Among these, microRNAs (miRNAs) are becoming increasingly important epigenetic biomarkers. These small (18–24 nucleotides (nts)), single-stranded, noncoding RNAs play critical roles in a variety of cellular processes, including apoptosis, cell cycle, proliferation, differentiation, and angiogenesis, by simultaneously regulating the expression levels of several genes. Many studies in CRC have revealed that miRNAs may function as oncogenes or tumor suppressors^4^ and play a key role in tumorigenesis, and their markedly changing expression profiles between normal and tumor tissues may be associated with metastatic processes and drug resistance. The miRNAs thus continue to gain prominence as biomarkers with great diagnostic, prognostic, and therapeutic potential.^1,3,5^

Furthermore, *N*^6^-methyladenosine (m6A) in RNA, considered the most abundant RNA methylation (∼50% of total methylated ribonucleotides), is a dynamic and reversible modification mediated by writer (m6A-methyltransferases), removed by eraser (demethylases), and recognized by reader (m6A-binding) proteins.^6−8^ The m6A RNA methylation is widely found in mRNAs and long noncoding RNAs (lncRNAs), and recent evidence suggests its role in variety of cancers, including CRC development, carcinogenesis, progression, and therapeutics.^2,9−14^

However, most of the research has historically relied on the expression levels of miRNAs and not on their methylation status to determine their biological significance. Although the exact mechanism underlying miRNA dysregulation in cancer remains to be elucidated, several studies have shown that epigenetic mechanisms play an important role in regulating miRNA expression, particularly DNA methylation of CpG islands located in the promoter regions, a biological process that adds methyl groups (CH_3_) to the C_5_ position of the cytosine ring, thus producing 5-methylcytosine (5-mC). Alterations in these mechanisms could perturb expression of miRNAs, subsequently altering gene and protein expression, leading to cancer progression.

Moreover, miRNA methylation profiles remain unknown in CRC patients, despite large-scale analyses of epigenetic alterations that are considered as the next big paradigm shift and are considered potentially better than somatic mutations for early detection and accurate classification of CRC.^3^ In this interesting and urgent scenario, recent but very scarce reports have suggested using miRNA methylation rather than their expression level for cancer diagnosis.^15^ However, these studies are hampered by a lack of sensitive detection strategies, which represent a bottleneck in deciphering the function of these modifications in miRNAs.^16^

Recently, high-throughput nucleic acid sequencing, borohydride reduction,^16^ and nontargeted mass spectrometry^15^ have been used to detect methylations in miRNAs. However, these technologies require expensive and high-maintenance instrumentation as well as expert technical knowledge for the interpretation of the results, which greatly limits their clinical implementation. It is important to highlight at this point that electrochemical bioplatforms have demonstrated great versatility and potential for the determination of miRNAs^17,18^ and methylations in nucleic acids.^19,20^ However, so far, they have not been developed to determine methylations in miRNAs. Therefore, being aware of this relevant unexplored niche and taking advantage of our previous experience in the development of competitive strategies for the determination of miRNAs^21−24^ and of m6A in total RNA,^25,26^ we have combined them in an innovative way to propose in this work the first electrochemical bioplatforms reported so far to determine both the total miRNA (regardless of its methylation) and its m6A methylation. The miRNA let-7a, one of the 9 members of the let-7 family, was selected due to its involvement in all CRC stages.^27−29^

## Experimental Section

The used apparatuses, instrument electrodes, reagents, and solutions and the analysis of cultured cells and tissue samples from CRC patients are described in detail in the Supporting Information.

### Bioconjugate Assembly on Magnetic Beads

Two approaches were optimized. The first one was designed for determination of the total content of the target miRNA regardless of its methylation status (miRNA let-7a). A second strategy was optimized for the determination of the target miRNA that is methylated with m6A (m6A-miRNA let-7a). Although the protocols for MBs modification were similar and involved the same type and volume of commercial MBs suspension, they differed in the number of assay steps. The implementation of the bioassays on the MBs required different incubation and washing steps in 1.5 mL microcentrifuge tubes. The incubation steps were carried out with 25 μL of the corresponding solution in an incubator shaker for a given time and under constant stirring and temperature (950 rpm, 37 °C). The washing steps were performed by manually shaking with 50 μL of the corresponding solution. After each incubation and washing step, the microcentrifuge tube was placed in an MBs concentrator for at least 3 min to remove the supernatant without MBs loss.

Briefly and for each determination, a 5 μL aliquot of the commercial vortex-homogenized suspension of Strep-MBs was placed in a 1.5 mL microcentrifuge tube and washed twice with 50 μL of B&W buffer or PBS for the determination of total miRNA let-7a or m6A-miRNA let-7a, respectively. Then, to determine total miRNA let-7a, MBs were incubated sequentially with a 0.05 μM bCp-15 solution (prepared in B&W) for 15 min and with the miRNA let-7a target solution (or 100 ng of total RNA extract) prepared in PBS for 30 min. A duplicate washing step with PBS was made between these two incubation steps. Thereafter, the bCp/miRNA let-7a/MBs were incubated for 30 min in a mixture solution, prepared in BB, containing 1.0 μg mL^–1^ anti-DNA/RNA Ab and 1/1000 diluted HRP-anti-mIgG Ab, with two final washings with 50 μL of BB after incubation.

Unlike the former protocol, the determination of m6A-miRNA let-7a required only one incubation step for both hybridization and capture of the bCp/m6A-miRNA let-7a heterohybrid on Strep-MBs. This was accomplished by incubating the Strep-MBs with a mixture solution prepared in PBS (pH 7.5) containing 0.05 μM bCp-15 and the m6A-miRNA let-7a target solution (or 100 ng of total RNA extract) for 30 min. In the last step and after a double washing with BB, the bCp/m6A-miRNA let-7a/MBs were incubated for 30 min in a mixture solution prepared in BB containing 1/2500 diluted anti-m6A Ab and 1/250 diluted HRP-anti-rIgG Ab, with two washings with BB after incubation.

The same protocol was used when Neu-MBs were employed but with 4 μL of the commercial Neu-MBs suspension.

### Amperometric Measurements

All amperometric measurements were performed at room temperature. For each measurement, a new SPCE/SP_4_CEs was placed in the PMMA casing with the Nd magnet/s located just below the working electrode (WE)/s of the SPCE/SP_4_CEs. The MBs modified as described in the previous section were resuspended in 50 or 5 μL of phosphate buffer (0.05 M, pH 6.0) and deposited on the surface of the WE/s of SPCE and SP_4_CEs, respectively.

The casing/SPCE-MBs and casing/SP_4_CEs-MBs assemblies, connected to the potentiostat via specific cables, were immersed into an electrochemical cell containing 10 or 20 mL of a freshly prepared solution of phosphate buffer (0.05 M, pH 6.0) supplemented with 1.0 mM HQ, respectively. Under continuous mechanical stirring, a constant potential of −0.20 V vs the Ag/AgCl electrode was applied. Once the background current was stabilized, 50 μL (single determination) or 100 μL (quadruple determination) of H_2_O_2_ solution (0.1 M) was added to the electrochemical cell, and the variation in the resulting cathodic current of the WE/s was recorded until the steady state was reached (∼100 s).

The values of the amperometric signals given in the manuscript correspond to the difference between the steady-state and background currents measured after and before the addition of H_2_O_2_, respectively, and were the mean values of 3 independent measurements. The error bars plotted in the figures were estimated as three times the standard deviation of such replicates.

## Results and Discussion

### Assay Fundamentals and Evaluation of Key Variables

The developed bioplatforms for the determination of the target miRNA total content and the target miRNA with m6A methylation shared the same components, such as the Strep-MBs, a synthetic biotinylated DNA probe complementary to a region of the target miRNA, selective detector antibodies marked with HRP-labeled secondary antibodies, and SPCEs to perform the electrochemical transduction. The main difference lies in the selection of the antibodies. While the determination of total miRNA required an antibody able to recognize epitopes of approximately 6 nts in DNA/RNA heterohybrids regardless of their sequence or methylation status (anti-DNA/RNA Ab),^30^ the determination of the m6A-methylated miRNA relied on a selective antibody to this m6A epimark (anti-m6A Ab).

**Figure 1:**
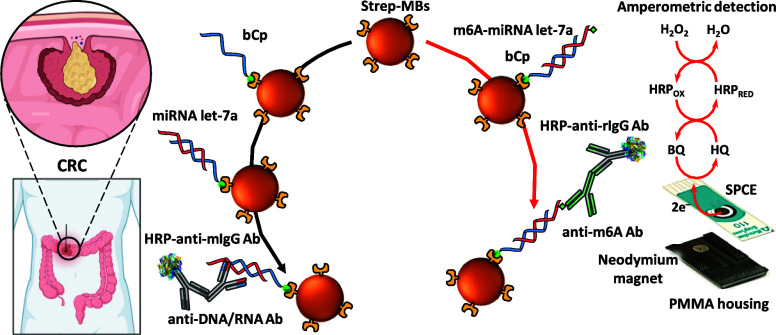
Schematic diagram showing the preparation of the developed bioplatforms for *in vitro* determination of the total content of the target miRNA regardless of its methylation and for the determination of the m6A-methylated target miRNA from tumor cells and tissues, using MBs and amperometric transduction on SPCEs with the HRP/H_2_O_2_/HQ system.

According to the results obtained in exhaustive optimization studies that will be discussed in more detail below, as shown in Figure 1, the determination of the total content of the target miRNA involved its capture on bCp-15/MBs by heterogeneous hybridization. However, the determination of the m6A-methylated target miRNA was accomplished by homogeneous biotinylated DNA/miRNA hybridization and capture on the surface of Strep-MBs. In both cases, the captured heterohybrids were enzymatically labeled with a mixture of detector and HRP-conjugated secondary antibodies. The resulting magnetic bioconjugates, prepared in 3 or 2 incubation steps and in 75 or 60 min for total miRNA let-7a and m6A-miRNA let-7a, respectively, were trapped on the WE surface of an SPCE, and amperometric transduction was performed in the presence of the HRP/H_2_O_2_/HQ system^31^ as indicated in the section “Amperometric Measurements”. The resulting cathodic current variation was proportional to the concentration of total miRNA let-7a or m6A-miRNA let-7a.

To fine-tune the bioplatforms for determination of the target miRNA total content or the target miRNA with m6A methylation, the key experimental variables were evaluated. These included, for instance, the protocol to carry out the determination, the length and concentration of the bCp, the incubation time of the bCp and the target miRNA for hybridization and capture of the resultant biotinylated DNA/RNA heterohybrids on the Strep-MBs, the concentration of the detector antibodies and HRP secondary antibodies, and the incubation time of their mixture with the bCp/miRNA let-7a/MBs and bCp/m6A-miRNA let-7a/MBs for the recognition and enzymatic labeling of the heterohybrids and the m6A, respectively. The results obtained are shown in Figures S1–S4 (in the Supporting Information). The influence of all of these variables was evaluated in a univariate manner by comparing the responses provided by the bioplatforms in the absence (B, blank) and in the presence (S, sample) of the indicated concentration of synthetic total miRNA let-7a and m6A-miRNA let-7a and considering the best S/B ratio value as a selection criterion.

First, the use of MBs modified with streptavidin (Strep-MBs) or neutravidin (Neu-MBs) as solid supports was compared. A remarkably larger S/B ratio was observed by using Strep-MBs (results not shown), which led us to select Strep-MBs for the development of the bioplatforms.

The influence of the bCp length is displayed in Figure S1 in the Supporting Information. Depending on the size, the capture probes hybridize either partially (bCp-15) or completely (bCp-22) with the target miRNA, having opposite effects on the determination of the target miRNA total content or the target miRNA with m6A methylation. These results were attributed to the characteristics of the detector antibodies. The anti-DNA/RNA Ab can recognize a 6 nts epitope in any DNA/RNA heterohybrid regardless of the sequence, which allows more molecules of this antibody to bind a longer heterohybrid. This gave rise to an amplification factor by also binding more secondary antibody molecules (HRP-anti-mIgG Ab) per heterohybrid when the longer bCp was used.^30,32^ However, the antibody used to detect the m6A epimark (anti-m6A Ab) reacts preferably with unpaired *N*^6^-methyladenosine both in DNA and in RNA, which justifies that a higher S/B was obtained with the shorter bCp leaving the m6A unpaired, as well as that it was not possible to detect m6A (S/B < 1) when the entire miRNA sequence hybridized to bCp-22. This result agrees with previous studies that report steric hindrance for antibody binding when a complementary probe is very close to the target m6A.^33,34^

An interesting test to demonstrate clinical usefulness is to investigate the effect of the bCp length on the amperometric responses provided by the bioplatform for the determination of the total miRNA amount when mixtures containing different ratios of miRNA let-7a and m6A-miRNA let-7a and using both capture probes were assayed (Figure S2 in the Supporting Information).

Figure S2 shows that the amperometric signals obtained using the shorter capture probe (bCp-15) were affected only when the amount of m6A-miRNA let-7a was higher than 10% (Figure S2a). However, no change in the amperometric responses with the amount of m6A-miRNA let-7a in the mixture was observed when the longer capture probe (bCp-22) was used (Figure S2b).

It is important to note that m6A methylation is confined to the hybrid only when bCp-22 is used. These results can be explained by the widely reported phenomenon of a spring-loaded base modification. The 6-methyl group within an adenine base exhibits two conformations, with the *syn* conformation being thermodynamically favored and therefore the most abundant. However, to hybridize, the m6A group must rotate to isomerize into a less stable conformation (*trans*), which hinders and slows hybridization kinetics.^33,35,36^ Accordingly, the destabilization of the DNA/RNA hybrid is higher when using bCp-22. In view of these results and to avoid the greater reduction in amperometric signal observed using the long bCp for total miRNA let-7a when the amount of m6A-miRNA let-7a was higher than 10% (values bars 100/0 and 90/10 in Figure S2a,b), bCp-15 was selected for further experiments.

Nevertheless, according to Konno et al.,^15^ the fraction of miRNA let-7a with m6A methylation at position 19 of its sequence in healthy and CRC-matched tumor tissues is in the range between 2 and 4%, respectively. Therefore, this reported low percentage allows us to ensure that with the developed bioplatforms, the determination of the total let-7a miRNA content will not be affected by the presence of its m6A fraction in these particular real biological scenarios.

The obtained results show that the length of the capture probes is a variable, which should be considered for the determination of each target miRNA, and that its selection will depend on both the required sensitivity (total amount) and the position of the methylated base.

Regarding the assay protocol for miRNA let-7a and m6A-miRNA let-7a, different procedures involving different 30 min incubation steps were tested, as detailed in Table S2 (in the Supporting Information).

The results shown in panels (a) of Figures S3 and S4 (in the Supporting Information) indicated that protocols 3A and 2B provided better results for the determination of the total target miRNA content and the m6A-methylated target miRNA, respectively, and therefore, these protocols were selected for the preparation of the bioplatforms. Importantly, in view of an eventual future implementation in a commercial device, since the sensitivity for the determination of the target miRNA total content was relatively high, a unified protocol 2B for the determination of both the total and the m6A-methylated target miRNA would be feasible.

The optimization studies regarding the concentrations of the bCp and the involved antibodies as well as the incubation times reflected the usual pattern with increasing S/B ratios until reaching a maximum from which they decreased due to agglutination phenomena or a lower efficiency of affinity reactions.

Table S3 (in the Supporting Information) summarizes all of the tested variables and the final values selected for the development of the bioplatforms.

### Analytical and Operational Characteristics

**Figure 2:**
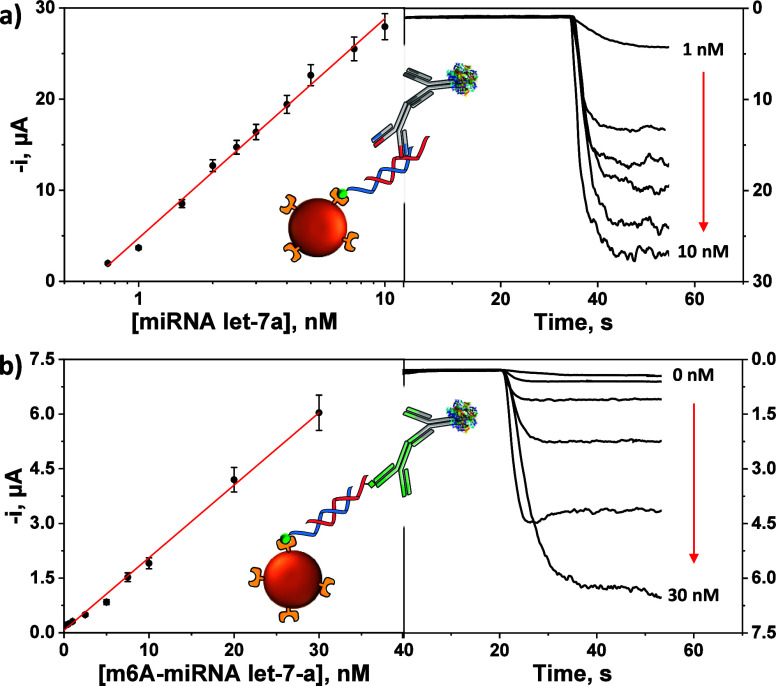
Calibration graphs and real amperometric traces obtained with the developed bioplatforms for the determination of synthetic total miRNA let-7a (a) and m6A-miRNA let-7a (b).

Under the selected experimental conditions, the analytical and operational characteristics of the developed bioplatforms were evaluated. The calibration plots shown in Figure 2 exhibited a semilogarithmic and linear behavior with the concentration of the unmethylated (a) and m6A-methylated (b) target miRNA, respectively. The analytical characteristics are summarized in Table 1.

**Table 1:** Analytical and Operational Characteristics Achieved by the Developed Bioplatforms for the Amperometric Determination of Synthetic Total miRNA let-7a and m6A-miRNA let-7a

parameter	total	m6A-methylated
linear dependence	*–i* vs log[miRNA let-7a]	–*i* vs [m6A-miRNA let-7a]
linear range, nM	0.28–10	0.48–30
slope	(24092 ± 1496) nA	(199 ± 10) nA nM^–1^
intercept	(4742 ± 838) nA	(70 ± 122) nA
*R*^2^	0.9935	0.9957
LOD, nM^a^	0.08	0.14
RSD_(*n* = 10)_, %	5.1	8.0
assay time, min	75	60

a Estimated as 3 × *s*_b_/slope (*s*_b_: standard deviation of 10 amperometric signals measured in the absence of the synthetic target miRNA).

The developed bioplatform allowed the determination of the total miRNA let-7a at the pM level. Importantly, the reached LOD value could be significantly improved by using a longer capture probe (S/B values of 45.8 vs 10.7 for bCp-22 and bCp-15, respectively, Figure S1a in the Supporting Information).

Only few reported methodologies can be directly compared with the bioplatform proposed in this work because they were either used for the determination of miRNAs^22^ or m6A in total RNA,^25,26^ but not for the determination of both at the same time. Compared with the method reported for the determination of total miRNA-21,^22^ which used a similar direct DNA/miRNA hybridization implemented on the surface of Strep-MBs and the recognition of the heterohybrid with anti-DNA/RNA, this involved enzymatic labeling with ProtA-polyHRP_40_. Such a strategy provided a lower LOD than that achieved in our work for the determination of total miRNA let-7a (0.4 vs 80 pM), which is attributed to the use of a longer bCp and a multienzyme reagent for HRP marking. Nevertheless, the achieved sensitivity is still compatible with the relevant applications. In addition, the methodology described in our work would not exhibit the expected problems in the application of the work reported in ref (22) considering the affinity of the bacterial protein used for enzymatic labeling (ProtA-polyHRP_40_) for mammalian immunoglobulins.^37^ This would be particularly relevant for the analysis of liquid biopsy samples such as serum or plasma with abundant concentrations of human IgGs that could sequester ProtA-polyHRP_40_, hindering the enzymatic labeling of the anti-DNA/RNA Ab.

Regarding the m6A methylation, the previously reported works^25,26^ aimed to determine the m6A modification at a global level in RNAs, but not regionally in miRNAs. Moreover, they exploited a completely different assay format (a direct competitive immunoassay in which anti-m6A is used as a capture bioreceptor and not as a detector element) so that the usefulness of a direct comparison is questionable. Indeed, the design used in this research, combining in a noncompetitive format the selectivity of a capture DNA probe complementary to a target miRNA region flanking the methylation position and a selective antibody to the target epimark, makes this bioplatform easily implementable with that used for the determination of total miRNA into a biotool that would allow the simultaneous determination of both the total miRNA and the m6A-methylated target.

It is also important to remark that the developed strategy can provide comparable sensitivities for the determination of the total content of different miRNAs (for instance, S/B ratios of 45.8 and 46.0 for 0.75 nM miRNA let-7a and miRNA-17, respectively).

### Selectivity

**Figure 3:**
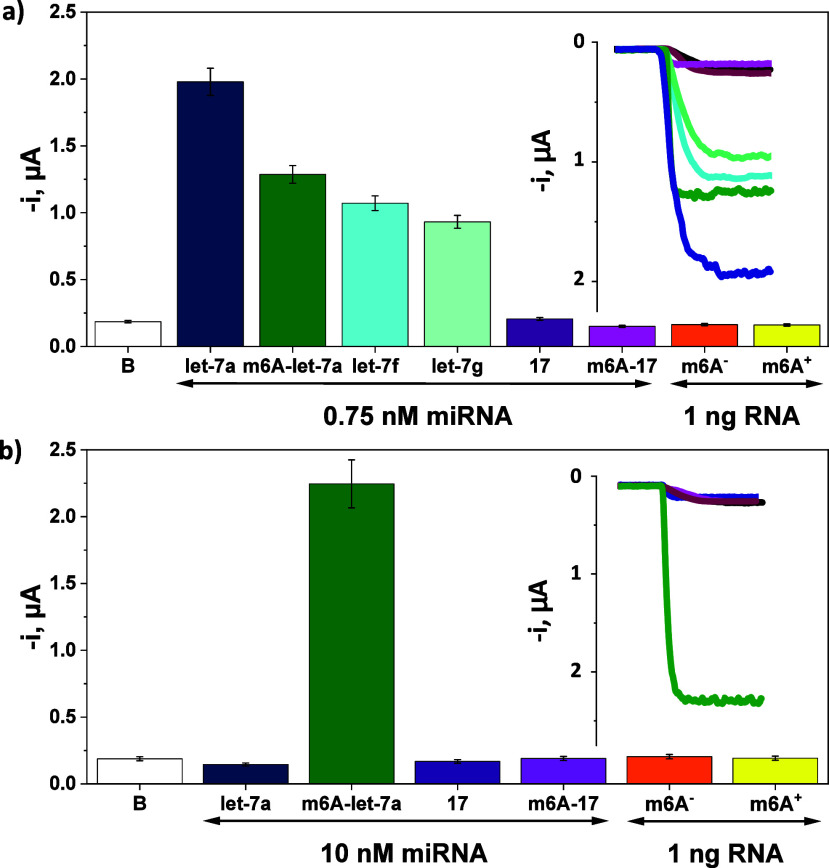
Amperometric responses and real traces provided by the developed bioplatforms for the determination of synthetic (a) total miRNA let-7a and (b) m6A-miRNA let-7a. Data are shown for the blank sample (no target, B), for synthetic target miRNA (miRNA let-7a) and nontarget miRNA (miRNA-17), in both unmethylated and m6A-methylated variants, two homologous sequences of the miRNA let-7a family (miRNAs let-7f and let-7g), and commercial unmethylated (m6A^–^) and 100% m6A-methylated (m6A^+^) RNA controls.

The selectivity toward the selected targets was evaluated. The amperometric responses obtained in the absence of any miRNA (B) and in the presence of either fully complementary synthetic target miRNA let-7a or a nontarget miRNA (miRNA-17) were compared for both unmethylated and m6A-methylated variants. In addition to these two miRNAs, which were shown to have increased m6A methylation in CRC patients,^15^ commercial fully unmethylated (m6A^–^) and 100% m6A-methylated (m6A^+^) control RNAs were assayed. The results shown in Figure 3 indicated that the bioplatform developed for the determination of the total content of the target miRNA provided an amperometric response significantly larger than that of the blank in the presence of both synthetic unmethylated and m6A-methylated target miRNAs (Figure 3a). However, the bioplatform developed for the determination of the m6A-methylated miRNA provided a significant amperometric response only in the presence of the m6A-methylated synthetic target miRNA (Figure 3b).

Moreover, considering the high homology between members of the miRNA let-7 family,^38^ additional experiments comparing the amperometric responses provided by the bioplatform for the determination of total miRNA let-7a against two other homologous miRNAs from the same family (let-7f and let-7g, differing in one and two nucleotides as compared to let-7a, respectively) were performed. The results in Figure 3a demonstrate that when using the shorter bCp-15, the miRNAs let-7f and let-7g gave 54 and 47% of the response provided by the target let-7a miRNA, respectively. The superior differences between target and mismatched miRNAs are attributed in this case to the fact that although both miRNAs let-7f and let-7g have only one unpaired base with bCp-15, the mismatches are located close to the 5′-end of the bCp and are thus easier to discriminate. Indeed, in analogous experiments carried out using bCp-22 instead of bCp-15, the miRNAs let-7f and let-7g gave 87 and 66% of the response provided by miRNA let-7a, respectively. This slightly lower discrimination ability of bCp-22 can be explained by a position of the mismatch in the middle of the miRNA-bCp duplex sequence, which is more difficult to recognize. These results thus confirm the selection of bCp-15 as a suitable capture probe to develop a more selective bioplatform.

This acceptable discrimination toward homologous miRNAs, even in nonstringent hybridization conditions, was similar to that previously reported for electrochemical biosensors involving the use of a synthetic DNA complementary probe and the same anti-DNA/RNA Ab.^21,22,24^

These results confirmed the excellent selectivity of the bioplatforms developed, attributable both to the selective hybridization of the target miRNA with bCp and to the specific recognition of the DNA/miRNA-formed heterohybrid by the anti-DNA/RNA Ab or to the m6A target methylation by the anti-m6A Ab. In fact, the selectivity of the anti-m6A Ab toward m6A methylation was also stated in one of our previous works analyzing synthetic oligomers containing a single 5-mC, 5-hmC, and m6A.^26^

### Analysis of Cultured Cancer Cells and Tissues from CRC Patients

The developed bioplatforms were employed to analyze the expression of miRNA let-7a and m6A-miRNA let-7a in total RNA extracted from cultured cells of different metastatic capacities and from the tissues of patients diagnosed with CRC at different stages.

**Figure 4:**
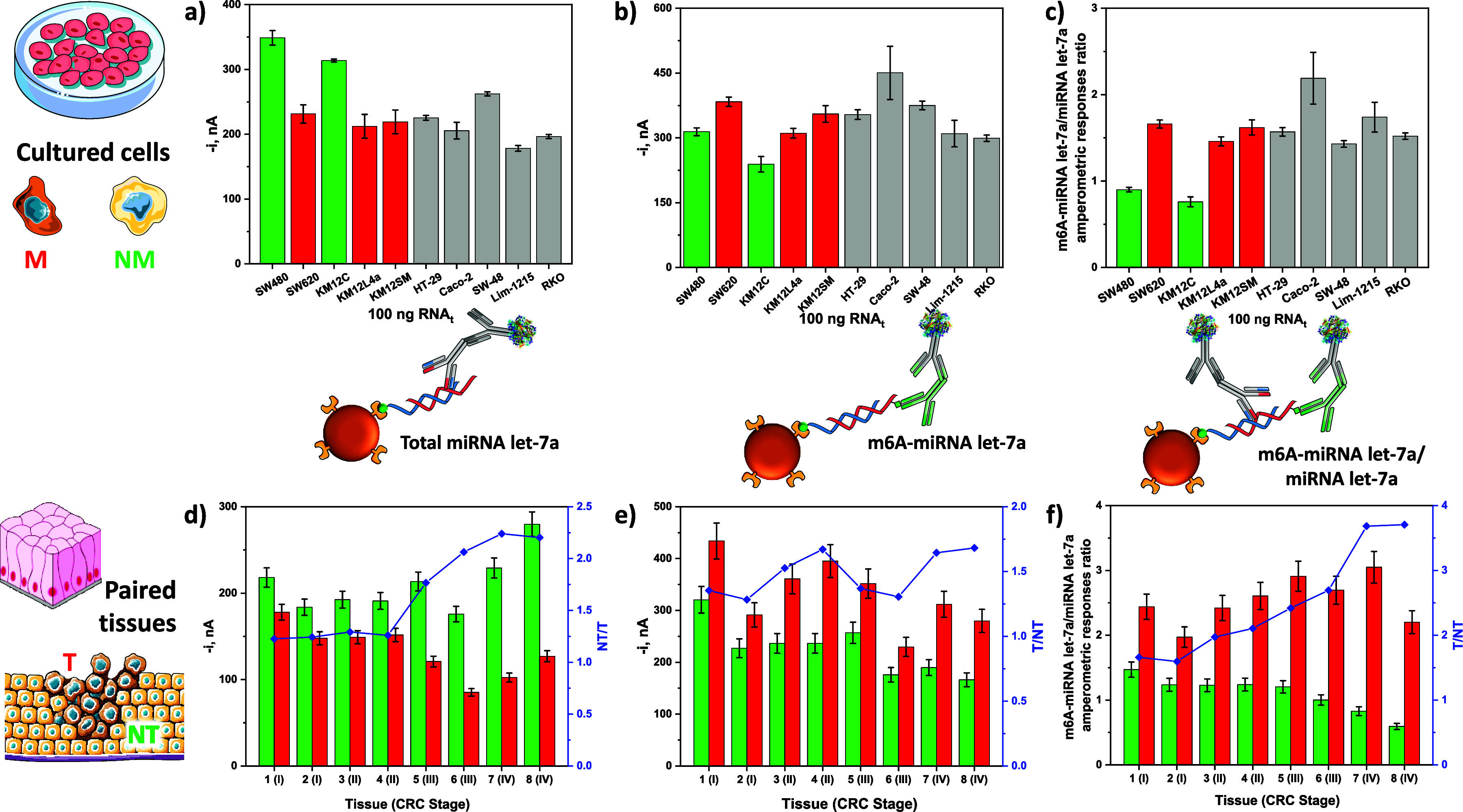
Comparison of the amperometric responses provided by the bioplatform for the determination of total miRNA let-7a (a,d) and m6A-miRNA let-7a (b,e) and ratio of the amperometric responses obtained with the bioplatform for m6A-miRNA let-7a and miRNA let-7a (c,f) for 100 ng of total RNA extracted from cultured cancer cells (a–c, green bars: isogenic nonmetastatic (NM) cell lines; red bars: isogenic metastatic (M) cell lines; gray bars: nonisogenic cell lines) and from tissues (tumor, T, and nontumor, NT, paired) of patients diagnosed with CRC at different stages (d–f, green bars: NT tissues; red bars: T tissues). NT/T or T/NT ratios are shown in blue.

The amperometric responses provided by the bioplatforms for 100 ng of total RNA extracted from different cancer cells and from paired normal and tumor tissues of CRC patients at different stages are shown in Figure 4. As can be seen, m6A methylation in the target miRNA was significantly increased both in cultured cancer cells with metastatic properties and in tumor tissues compared to paired normal tissues of CRC patients (Figure 4b,e). It is important to highlight that the results in tissues agreed with those previously reported by other authors who found increased m6A methylation in miRNA let-7a in pancreatic and CRC tissues compared to paired normal tissue samples using a nontargeted mass spectrometry sequencing technique.^15^ These results also agree with reports claiming increased m6A levels in total RNA extracted from CRC cells and tissues through the upregulation of the methyltransferase METTL3, which also promotes metastasis of CRC,^9,12^ and with the analysis of m6A-related RNAs other than miRNAs as prognostic factors in CRC.^13^ Furthermore, the results obtained in the determination of total miRNA let-7a in cultured cancer cells and tissues (Figure 4a,d) also agree with the downregulation of this miRNA in CRC tissues^27,29,39^ and the accumulating evidence indicating that let-7 miRNAs function as a tumor suppressor in CRC.^28^ Moreover, results were mostly consistent among CRC stages (Figure 4d,e), except for patients #5 and #6, which can be attributed to the individual heterogeneity among CRC tumors and CRC patients (Figure 4e). In fact, scatter plots comparing the amperometric responses provided by the bioplatforms for the determination of miRNA let-7a and m6A-miRNA let-7a for NT vs T tissues for all stages using the Wilcoxon matched-pairs signed-rank test (*t* test type) showed significant differences in the expression of total and m6A-methylated miRNA let-7a in both tissue types, with *p* values of 0.0078 in both cases (Figure S5 in the Supporting Information).

The methylation level of the target miRNA in cultured cells and CRC tissues was estimated as the ratio between the amperometric responses provided by the bioplatform for m6A-miRNA let-7a and miRNA let-7a (Figure 4c,f). Noticeably, despite the interindividual heterogeneity observed especially for patients #5 and #6, the ratio between m6A-miRNA let-7a and miRNA let-7a was increasing for tumor tissues from stage II to stage IV, while it decreased for nontumor tissues (Figure 4f). Hence, the overall ratio between tumor and nontumor tissues from CRC patients at stage II to stage IV clearly increased (Figure 4f). Indeed, the evaluation of the obtained results by means of the ROC curves (Figure S6 in the Supporting Information), whose most relevant parameters are summarized in Table 2, showed that such a ratio allowed discrimination of the metastatic capacities of cancer cells and identification of tumor tissues and their stage in CRC. These results agree with reports claiming enhanced m6A RNA methylation with the increasing CRC grade.^10,11^

**Table 2:** Characteristic Parameters of the ROC Curve Analysis for the Results Obtained with the Developed Bioplatforms for the Analysis of Total RNA Extracted from Cultured Cells and Tissues of CRC Patients

	cultured cells			human tissues	
signal m6A-miRNA let-7a/miRNA let-7a	SW480^a^/SW620^b^	KM12C^a^/KM12SM^b^	KM12C^a^/KM12L4a^b^	NT/T	early NT/T CRC (I–II)/advanced NT/T CRC (III–IV)
AUC (%)	100	100	100	100	100
sensitivity (%)	100	100	100	100	100
specificity (%)	100	100	100	100	100
cutoff	1.278	1.167	1.075	1.721	2.39

a Nonmetastatic.

b Metastatic. NT: nontumor. T: tumor.

Although clinical acceptance of miRNA methylation as a biomarker would require a larger-scale study with cancer patients and healthy controls, the results reported here provide evidence of the biological importance of RNA methylation status not only for diagnosis but also for CRC staging. It is important to mention that unlike the few studies reported that used high-throughput nucleic acid sequencing and nontargeted mass spectrometry, this work reports for the first time the possibility of providing such evidence with an electrochemical bioplatform in just 75 min. In addition, the developed bioplatforms are competitively advantageous in terms of cost, compatibility with multiplexed determinations, and applicability at the point of need.

**Figure 5:**
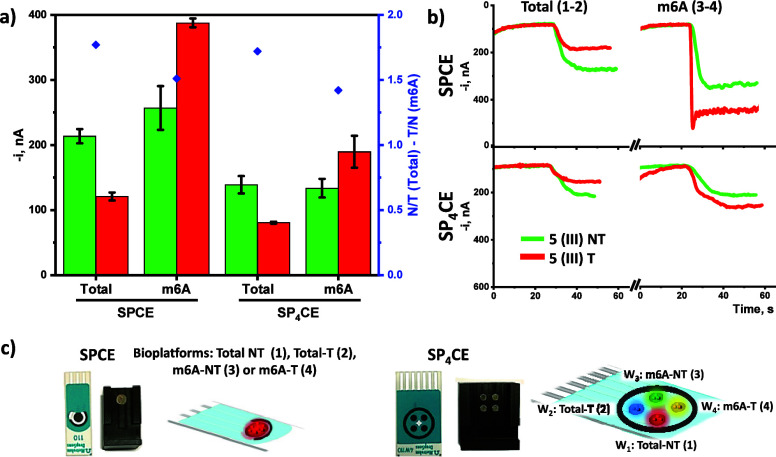
Comparison of the amperometric responses obtained for the single or quadruple determination of miRNA let-7a and m6A-miRNA let-7a in 100 ng of total RNA extracted from T and paired NT tissues from patient 5 (III) (a). Real amperometric traces (b). Images of the corresponding homemade magnetic holding blocks, SPCE and SP_4_CEs, and drawings with the type of MB captured on their surface to perform multiplexed determinations (c).

Once the applicability of the developed bioplatforms for the single determination of the total or m6A-methylated target miRNA in total RNA extracted from cells and tissues was demonstrated, the possibility of their simultaneous determination using a quadruple electrochemical detection bioplatform was evaluated. As a proof of concept, the analysis of paired healthy and tumor tissues of a patient diagnosed with advanced CRC (sample 5 (III) in Figure 4d,e) was carried out. Therefore, bioconjugates prepared as detailed in the section “Bioconjugate Assembly on Magnetic Beads” were captured onto SPCEs or SP_4_CEs to perform individual or quadruple amperometric transductions (section “Amperometric Measurements”), respectively. The results displayed in Figure 5 showed that the amperometric responses were comparable on both SPCEs and SP_4_CEs, with small differences in intensity attributable to the difference between the surface areas of the WEs.

It is important to mention that the multiplexing capacity of electrode arrays can be expanded to 8 even 96 electrochemical cells. Hence, together with the versatility of the developed bioplatforms to detect other miRNAs or other RNA methylations of relevance in CRC (e.g., 5-methylcytosine, 5-mC, *N*^1^-methyladenosine, m1A, and 7-methylguanosine, m7G), the total contents and the presence of methylation in different miRNAs in the same sample can be determined simply by changing the bCp and detection and HRP secondary antibodies. In addition, the same miRNA can be determined simultaneously in different samples. These potential abilities entail important advantages in terms of shorter overall analysis times, smaller sample volume, reduced cost, and more accurate diagnostics.^40^ In this context, the developed bioplatform may be envisaged as an attractive tool to shed light on the miRNA methylome and to identify new molecular signatures of methylated miRNAs with diagnostic, prognostic, and therapeutic value.

## Conclusions

The first electrochemical bioplatforms able to determine the total content of a target miRNA regardless of its methylation status and the total content of the methylated target miRNA are presented in this work. The developed bioplatforms involved homogeneous hybridization of the target miRNA with a synthetic biotinylated DNA probe, the capture of the formed DNA/miRNA heterohybrids on the surface of magnetic microcarriers, and their recognition with antibodies selective to these heterohybrids or to the target epimark for the determination of the total or m6A-methylated target miRNA, respectively, which were enzymatically labeled with HRP-conjugated secondary antibodies. In both cases, amperometric transduction was performed on the surface of disposable electrodes after the capture of the resulting HRP-marked bioconjugates. The developed bioplatforms exhibited attractive analytical and operational characteristics with assay times as short as 1 h using simple protocols. The bioplatforms were applied to the analysis of RNA samples extracted from cancer cells and paired normal and tumor tissues of patients diagnosed with CRC. This is the first-time application of electrochemical bioplatforms to simultaneously analyze miRNA expression and miRNA methylation, with the aim to discriminate the metastatic capacities of cancer cells and to identify tumor tissues and their stage in CRC, proving in a pioneering way the potential of the target miRNA m6A methylation to serve as a prognostic biomarker for CRC. It is important to highlight that the newly developed biotool does not require expensive and maintenance instrumentation as well as expert technical knowledge for the interpretation of results conversely to those used up to now for the detection of methylations in miRNAs. Moreover, due to the versatility and compatibility with multiplexed determinations that this type of electrochemical bioplatform possesses, it may be designed to simultaneously detect different types of methylation in the same miRNA or the same methylation in different miRNAs to shed light and empower the significance of miRNA methylation in the prognosis of cancer.

## Supporting Information Available

Used apparatuses, instrument electrodes, reagents, and solutions; synthetic oligonucleotides used (Table S1); analysis of cultured cells and tissue samples from CRC patients; effect of the capture probe length (Figures S1 and S2); protocols tested (Table S2); optimization of experimental variables (Figures S3 and S4 and Table S3); scatter plots showing results in clinical samples (Figure S5); and ROC curves analysis of the obtained results (Figure S6) (PDF)
