# Supplementary material for: Bringing to Light the Importance of the miRNA Methylome in Colorectal Cancer Prognosis Through Electrochemical Bioplatforms
Source: Anal Chem. 2024 Feb 13;96(11):4580–8. doi: 10.1021/acs.analchem.3c05474 (PMC10955513; doi:10.1021/acs.analchem.3c05474)
Supplement: Supplementary file 1 — ac3c05474_si_001.pdf [file ac3c05474_si_001.pdf]

SUPPORTING INFORMATION

Bringing to light the importance of miRNA methylome in colorectal cancer prognosis through electrochemical bioplatforms

Eloy Povedano[a], Víctor Ruiz-Valdepeñas Montiel[a], Ravery Sebuyoya[b,c], Rebeca M. Torrente-Rodríguez[a], Maria Garranzo-Asensio[d], Ana Montero-Calle[d], José M. Pingarrón[a], Rodrigo Barderas[d,*], Martin Bartosik[b,*], Susana Campuzano[a,*]

[a]*Departamento de Química Analítica, Facultad de CC. Químicas, Universidad Complutense de Madrid, Pza. de las Ciencias 2, 28040-Madrid, Spain*

[b]*Research Centre for Applied Molecular Oncology, Masaryk Memorial Cancer Institute, Zluty kopec 7, 656 53 Brno, Czech Republic*

[c]*National Centre for Biomolecular Research, Faculty of Science, Masaryk University, Kamenice 5, 625 00 Brno, Czech Republic*

[d]*Chronic Disease Programme, UFIEC, Institute of Health Carlos III, Majadahonda, 28220-Madrid. Spain*

*E-mail addresses: r.barderasm@isciii.es (R. Barderas); martin.bartosik@mou.cz (M. Bartosik); susanacr@quim.ucm.es (S. Campuzano)

CONTENTS	PAGE
**Apparatus, instruments and electrodes**	S3
**Reagents and solutions**	S3-S5
**Table S1**	S4
**Analysis of cultured cells and tissues samples from CRC patients**	S5
**Figure S1**	S6
**Figure S2**	S6
**Table S2**	S7
**Figure S3**	S8
**Figure S4**	S9
**Table S3**	S10
**Figure S5**	S11
**Figure S6**	S12
**References**	S13

Apparatus, instruments and electrodes.

Screen-printed carbon electrodes (SPCEs) with one carbon working electrode (WE) (SPCE, DRP110, 4 mm Ø), a carbon auxiliary electrode and a silver (Ag) pseudo-reference electrode with its corresponding specific cable connector (DRP-CAC) and quadruple screen-printed carbon electrodes (SP_4CEs) with four carbon working electrodes (WEs) (DRP-4W110, 2.95 mm Ø) with shared carbon auxiliary and Ag pseudo-reference electrodes and the specific cable connector (DRP-CONNECT4W) from Metrohm-DropSens (Spain), and a potentiostat (model 812B, CH Instruments, Austin, TX, controlled by the CHI812B software) were used for the electrochemical measurements. Laboratory-fabricated polymethyl methacrylate (PMMA) housing, with one or four built-in neodymium (Nd) magnets (AIMAN GZ), were used for reproducible and stable capture of the magnetic bioconjugates on the working electrode/s of the SPCEs and SP_4CEs, respectively. Other apparatus or instrumentation used included: a precision pH meter (Crison Basic 20+), a Vortex (Bunsen AGT-9), a steam sterilizer (Raypa), a biological safety cabinet (Telstar Biostar), an incubator shaker (Optic Ivymen® System, Comecta S.A., Sharlab), and a magnetic beads concentrator (DynaMag™-2, 123.21D, Invitrogen Dynal AS).

Reagents and solutions.

All used reagents were of the highest available analytical grade. Neutravidin (Neu) coated magnetic beads (MBs) (Neu-MBs, Ø = 1.0 μm, 10 mg mL^{-1}, Cat. No: GE78152104011150) were purchased from SpeedBeads™ GE Healthcare and streptavidin (Strep) modified MBs (Strep-MBs, Ø = 2.8 μm, 10 mg mL^{-1}, Cat. No: 11205D) were purchased from Invitrogen-ThermoFisher™. These commercial MBs have already been exhaustively characterized by the supplier company (https://www.sigmaaldrich.com/ES/es/product/sigma/ge78152104011150; https://www.thermofisher.com/order/catalog/product/11205D; https://www.thermofisher.com/document-connect/document-connect.html?url=https://assets.thermofisher.com/TFS-Assets%2FLSG%2Fmanuals%2FMAN0014017_Dynabeads_M280_Streptavidin_UG.pdf). N6-methyladenosine (m6A) Rabbit Polyclonal Antibody (anti-m6A Ab, Ref: A-1801), $m6A^{-}$ (RNA containing no m6A) and $m6A^{+}$ (RNA with 100 % of m6A) control RNAs were components of the EpiQuik™ m6A-RNA Methylation Quantification Colorimetric Kit (Cat. No: P-9005) purchased from Epigentek. Goat Polyclonal Antibody against Mouse Immunoglobulin (IgG) conjugated with HRP (HRP-anti-mIgG Ab, ab97265) and Goat anti-Rabbit IgG (H+L) conjugated with HRP (HRP-anti-rIgG Ab, Cat. No: 170-6515) were acquired

from Abcam and BioRad, respectively. Mouse anti-DNA/RNA hybrid clone S9.6 antibody (anti-DNA/RNA Ab, MABE 1095), hydroquinone (HQ), hydrogen peroxide (30 %, w/v) and ethylenediaminetetraacetic acid (EDTA) were purchased from Sigma-Aldrich. NaH2PO4, Na2HPO4, NaCl, KCl and Tris-HCl were purchased from Scharlab. A blocker casein solution (a ready-to-use, PBS solution of 1 % w/v purified casein, BB) from Thermo Fisher Scientific was also used. All used synthetic oligonucleotides (sequences in Table S1) were purchased from Integrated DNA Technologies or Sigma-Aldrich, reconstituted upon reception in sterilized H_2O to 100 μM and stored at −80 °C into small aliquots.

Table S1. Synthetic oligonucleotides used in this work.

Name	Sequence (5′→3′)	Company
15-nts b-DNA miRNA let-7a bCp (bCp-15)**	CAACCTACTACCTCA–biotin	Sigma-Aldrich
22-nts b-DNA miRNA let-7a bCp (bCp-22)**	AACTATACAACCTACTACCTCA–biotin	Sigma-Aldrich
miRNA let-7a*	UGAGGUAGUAGGUUG UAU**A**GUU	IDT Technologies
m6A-miRNA let-7a	UGAGGUAGUAGGUUGUAU**m6A**GUU	IDT Technologies
miRNA let-7f*	UGAGGUAGUAGAUUGUAU**A**GUU	Sigma-Aldrich
miRNA let-7g*	UGAGGUAGUAGUUUGUAC**A**GUU	Sigma-Aldrich
12-nts b-DNA miRNA-17 bCp**	GTAAGCACTTTG–biotin	Sigma-Aldrich
23-nts b-DNA miRNA-17 bCp**	CTACCTGCACTGTAAGCACTTTG–biotin	Sigma-Aldrich
m6A-miRNA-17	CAAAGUGCUUAC**m6A**GUGCAGGUAG	IDT Technologies
miRNA-17*	CAAAGUGCUUAC**A**GUGCAGGUAG	Sigma-Aldrich

b: biotin; m6A: N^6*-methyladenosine.*
In bold: unmethylated or methylated adenosine in the miRNA.
Underlined: mismatches bases.

*Unmethylated synthetic miRNAs.
**bCps were designed to be fully complementary to the entire sequence (bCp-22) or to only a portion of miRNA let-7a (bCp-15) such that the methylated base did not hybridize.
DNA bases are all deoxyribonucleotides and miRNA sequences are made of ribonucleotides.*

The following buffer solutions were prepared in Milli-Q water (18 MΩ cm at 25 °C): Binding and Washing buffer (B&W pH 7.5, consisting of 10 mM Tris-HCl, 1 mM EDTA and 2 M NaCl), phosphate buffer (0.05 M, pH 6.0) and PBS buffer (10 mM, pH 7.5). B&W and PBS buffer were sterilized before use.

Analysis of cultured cells and tissues samples from CRC patients.

HT-29, RKO, Caco-2, Lim1215 and SW480 and SW620 isogenic colorectal cancer cells were obtained from the ATCC cell repository. KM12C, KM12SM and KM12L4a isogenic colorectal cancer cells were from I. Fidler' laboratory, MD Anderson Cancer Center (Houston, TX). All cell lines were cultured according to established procedures.[1]

Tissue samples from CRC patients diagnosed with different stages (I-IV) were provided by the biobank of the Hospital Clínico San Carlos after approval of the Ethical Review Boards of this institution (CEI PI 45). These samples were reviewed by an experienced pathologist and 6-mm sections of paired normal and tumor colorectal tissues were sectioned in parallel during surgery, placed in vials and stored at −80 °C until total RNA extraction. Samples were used accomplishing all the ethical issues and relevant guidelines and regulations for sample handling and experiments performance. All individuals gave their written informed consent to participate in the study.

Total RNA was extracted from cultured cells and tissues using TRIzol reagent following the manufacturer's recommended protocol, and the concentration and purity of the total extracted RNA were determined with a NanoDrop ND-1000 spectrophotometer (Thermo Fisher Scientific).[2] Their quality was also evaluated on agarose gels prior further use.

The analysis of total RNA extracts was performed following the same protocol detailed for the synthetic targets in sections “Bioconjugates assembly on magnetic beads” and “Amperometric measurements” in the main text.

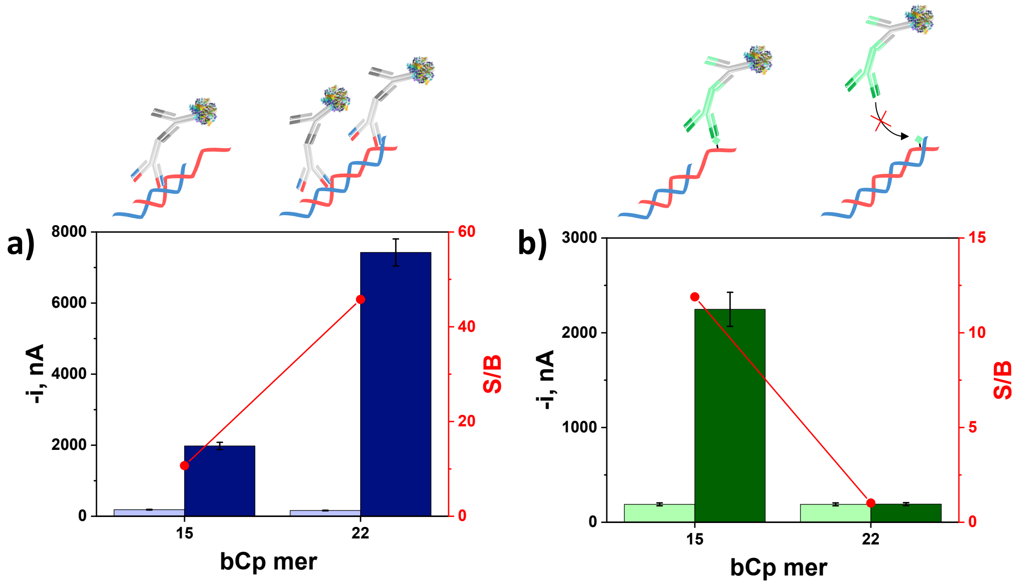

Figure S1. Dependence of the amperometric responses provided by the bioplatforms developed for the amperometric determination of synthetic total miRNA let-7a a) and m6A-miRNA let-7a b) on the length of the bCp used in the absence (light bars) and in the presence (dark bars) of 0.75 nM of synthetic total miRNA let-7a and 10 nM of m6A-miRNA let-7a as well as the values of the corresponding S/B ratio (in red).

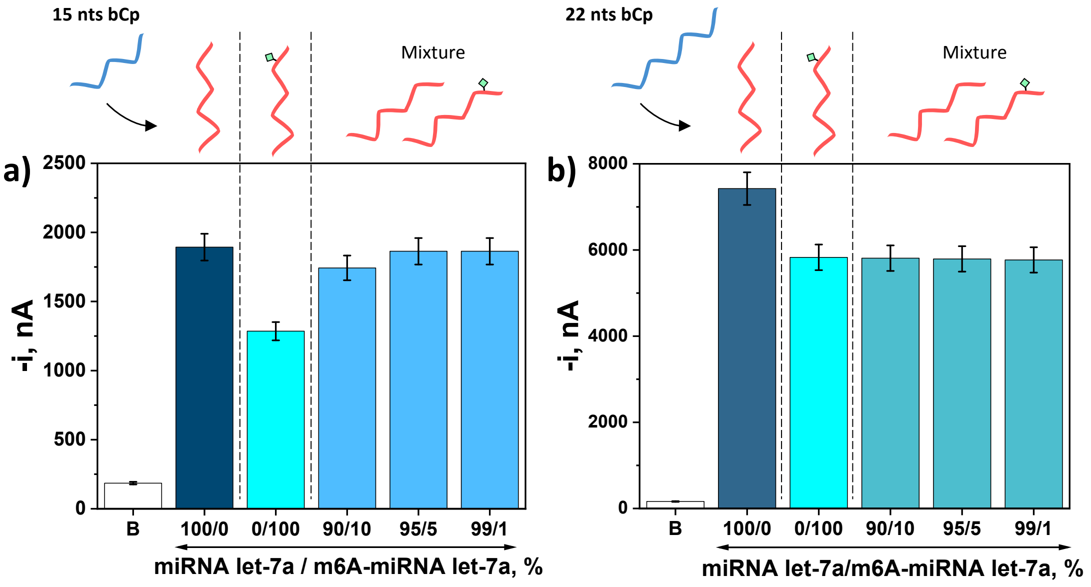

Figure S2. Amperometric responses provided by the bioplatform developed for the amperometric determination of total miRNA amount in the absence (B, white bar) and in the presence of 0.75 nM mixtures containing different ratios of miRNA let-7a and m6A-miRNA let-7a using bCp-15 a) and bCp-22 b).

Table S2. Protocols tested for the determination of total miRNA let-7a and m6A-miRNA let-7a.

<table>
<tr><th>Number of incubation steps</th><th>Protocol</th><th>Steps</th><th>Total time, min</th></tr>
<tr><td>1</td><td>1</td><td>bCp + Target + DAb + HRP-anti-IgG Ab</td><td>30</td></tr>
<tr><td rowspan="3">2</td><td>2A</td><td>i) bCp
ii) Target + DAb + HRP-anti-IgG Ab</td><td rowspan="3">60</td></tr>
<tr><td>2B</td><td>i) bCp + Target
ii) DAb + HRP-anti-IgG Ab</td></tr>
<tr><td>2C</td><td>i) bCp + Target + DAb
ii) HRP-anti-IgG Ab</td></tr>
<tr><td rowspan="3">3</td><td>3A</td><td>i) bCp
ii) Target
iii) DAb + HRP-anti-IgG Ab</td><td rowspan="3">90</td></tr>
<tr><td>3B</td><td>i) bCp + Target
ii) DAb
iii) HRP-anti-IgG Ab</td></tr>
<tr><td>3C</td><td>i) bCp
ii) Target + DAb
iii) HRP-anti-IgG Ab</td></tr>
<tr><td>4</td><td>4</td><td>i) bCp
ii) Target
iii) DAb
iv) HRP-anti-IgG Ab</td><td>120</td></tr>
</table>

**DAb: anti-DNA/RNA Ab or anti-m6A Ab.*
***HRP-anti-IgG Ab: HRP-anti-mIgG Ab or HRP-anti-rIgG Ab.*

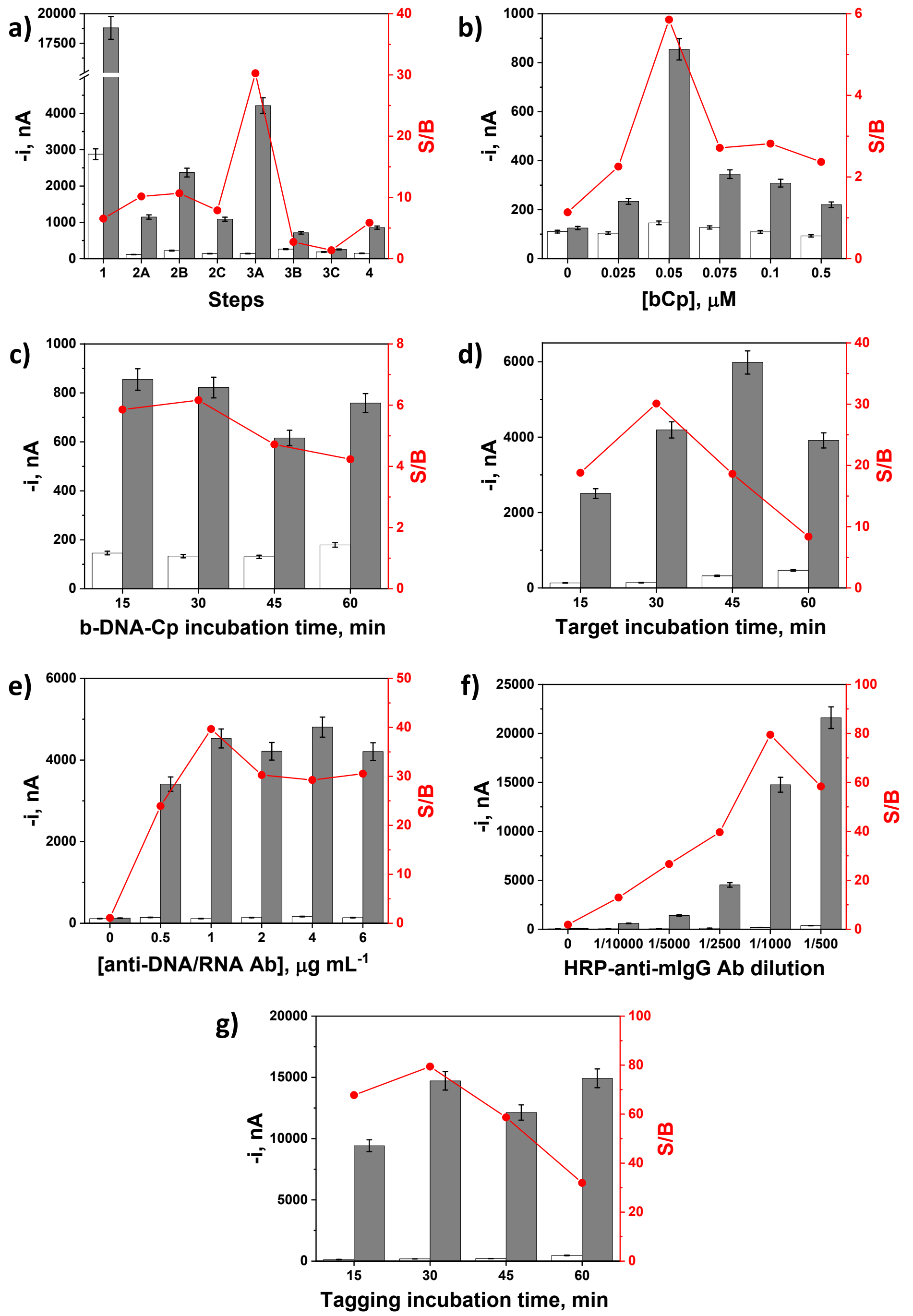

Figure S3. Dependence of the amperometric responses obtained with the developed bioplatform for the determination of total miRNA amount in the absence (B, white bars) and in the presence (S, grey bars) of 2.5 nM synthetic total miRNA let-7a as well as the values of the corresponding S/B ratio with: number of steps used in the preparation of the bioconjugates a), bCp concentration b), incubation time with bCp c), incubation time with the target miRNA d), concentration of anti-DNA/RNA Ab e), dilution of HRP-anti-mIgG Ab f) and incubation time with the anti-DNA/RNA Ab and HRP-anti-mIgG Ab mixture g). S/B ratios are shown in red.

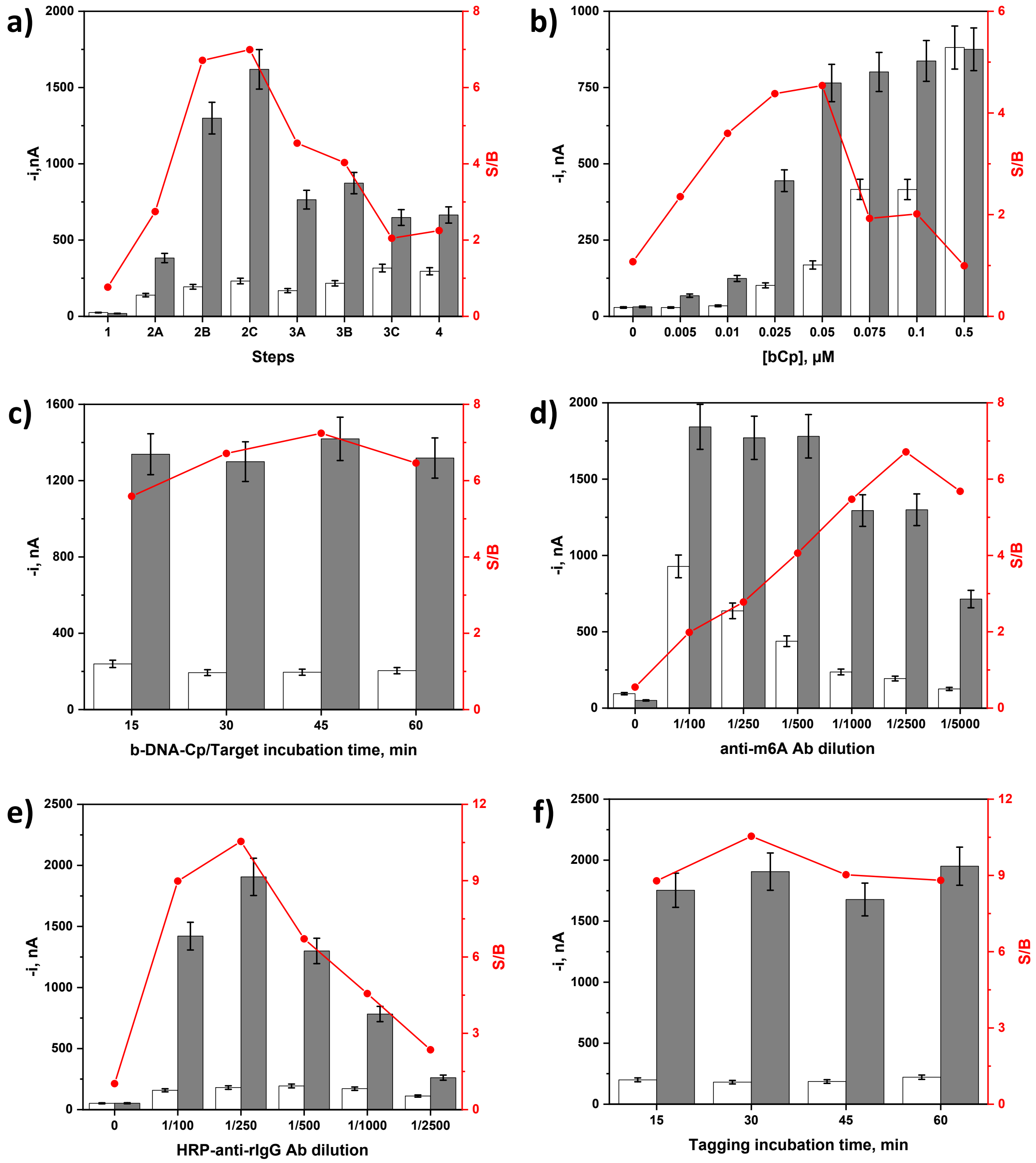

Figure S4. Dependence of the amperometric responses provided with the developed bioplatform for the determination of m6A-miRNA let-7a in the absence (B, white bars) and in the presence (S, grey bars) of 10 nM synthetic m6A-miRNA let-7a as well as the values of the corresponding S/B ratio with: number of steps involved in the preparation of the bioconjugates a), bCp concentration b), incubation time with the bCp + m6A-miRNA let-7a mixture c), dilution of anti-m6A Ab d), dilution of HRP-anti-rIgG Ab e) and incubation time with the anti-m6A Ab and HRP-anti-rIgG Ab mixture f). S/B ratios are shown in red.

Table S3. Experimental variables tested and selected in the development of the bioplatforms for the amperometric determination of total miRNA let-7a and m6A-miRNA let-7a.

Variable	Type/Range tested	Total miRNA	m6A-methylated
MBs type	Strep-MBs and Neu-MBs	Strep-MBs	
bCp length, nts	15, 22	15	
[bCp], μM	0.0–0.5	0.05	
Protocol	1, 2A-C, 3A-C, 4	3A	2B
bCp incubation time, min	15–60	15	--
Target incubation time, min	15–60	30	--
[anti-DNA/RNA Ab], μg mL^{-1}	0.0–6.0	1.0	--
HRP-anti-mIgG Ab dilution	0–1/500	1/1000	--
bCp + Target mixture incubation time, min	15–60	--	30
anti-m6A Ab dilution	0–1/100	--	1/2500
HRP-anti-rIgG Ab dilution	0–1/100	--	1/250
Tagging incubation time, min	15–60	30	

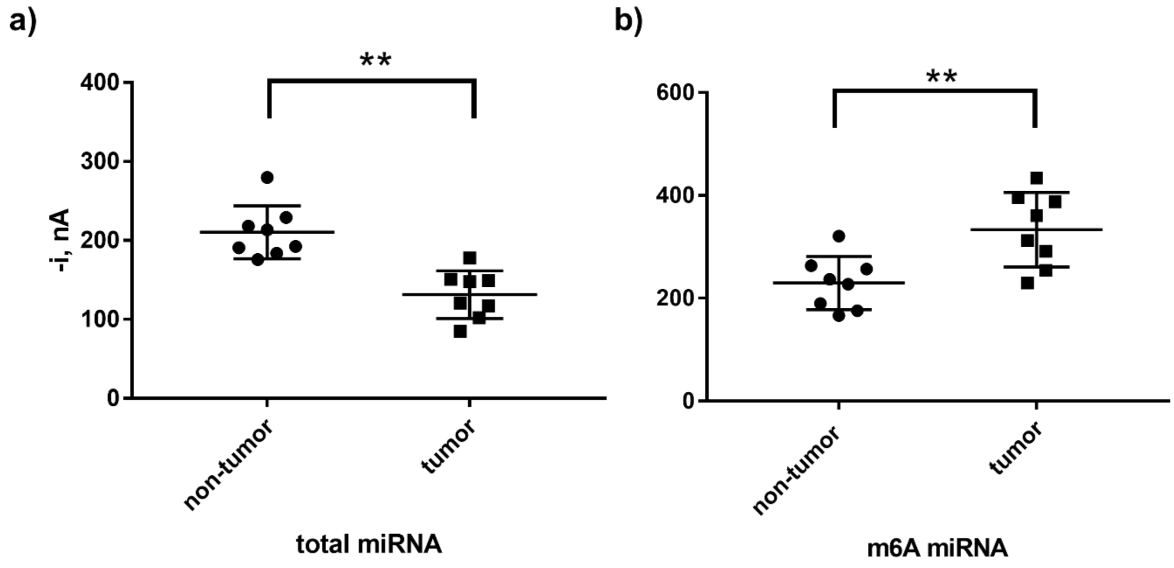

Figure S5. Scatter plots showing mean amperometric signals of eight paired clinical samples (NT *vs.* T) measured with the developed bioplaforms for total miRNA content a) and m6A methylated miRNA content b). Two asterisks denote significant differences between these two groups (p-value < 0.01). P-values were calculated using the Wilcoxon matched-pairs signed rank test.

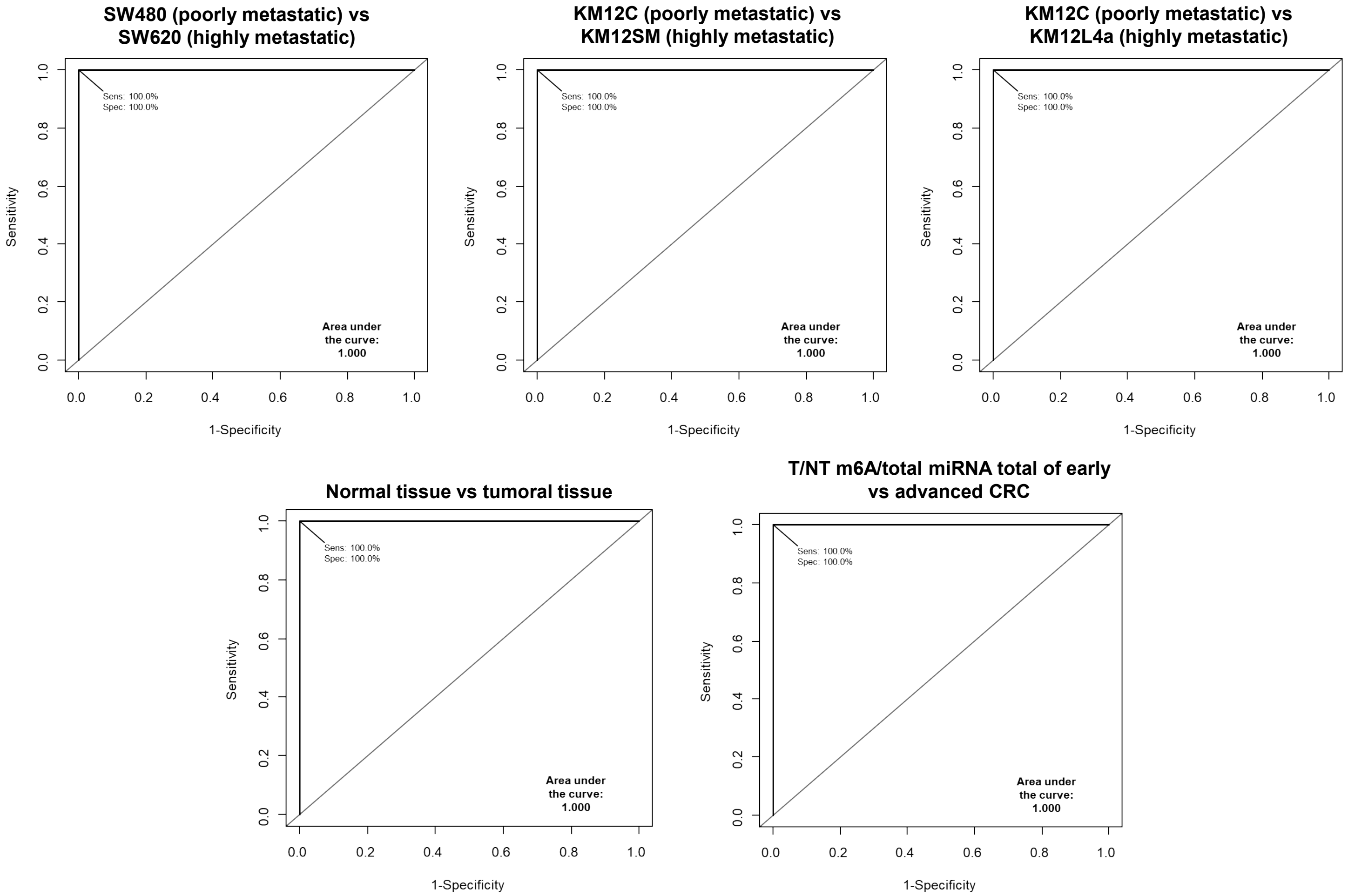

Figure S6. ROC curve analyses for the discrimination of the indicated groups according to the data displayed in **Figure 4** (main text). Sensitivity and specificity, and area under the curve are also depicted.

References

[1] Torrente-Rodríguez, R. M.; Ruiz-Valdepeñas Montiel, V.; Campuzano, S.; Pedrero, M.; Farchado, M.; Vargas, E.; Manuel de Villena, F. J.; Garranzo-Asensio, M.; Barderas, R.; Pingarrón, J. M. Electrochemical sensor for rapid determination of fibroblast growth factor receptor 4 in raw cancer cell lysates. *PloS One* **2017**, *12*, e0175056.

[2] Garranzo-Asensio, M.; San Segundo-Acosta, P.; Povés, C.; Fernández-Aceñero, M. J.; Martínez-Useros, J.; Montero-Calle, A.; Solís-Fernández, G.; Sánchez-Martínez, M.; Rodríguez, N.; Cerón, M. A.; Fernández-Díez, S.; Domínguez, G.; de los Ríos, V.; Peláez-García, A.; Guzmán-Aranguez, A.; Barderas, R. Identification of tumor-associated antigens with diagnostic ability of colorectal cancer by indepth immunomic and seroproteomic analysis. *J. Proteomics* **2020**, *214*, 103635.
